# Motion-corrected multiparametric renal arterial spin labelling at 3 T: reproducibility and effect of vasodilator challenge

**DOI:** 10.1007/s00330-018-5628-3

**Published:** 2018-07-10

**Authors:** Saba Shirvani, Paweł Tokarczuk, Ben Statton, Marina Quinlan, Alaine Berry, James Tomlinson, Peter Weale, Bernd Kühn, Declan P. O’Regan

**Affiliations:** 1 Medical Research Council (MRC), London Institute of Medical Sciences (LMS), Imperial College London, Hammersmith Hospital Campus, Du Cane Road, London, W12 0NN UK; 2 Department of Chemistry, Imperial College London, South Kensington Campus, Exhibition Road, London, UK; 3 Siemens Healthcare Ltd, Frimley, UK; 4 Siemens Healthcare GmbH, Erlangen, Germany

**Keywords:** Blood flow velocity, Computer-assisted image processing, Magnetic resonance imaging, Renal circulation, Vasodilator agents

## Abstract

**Objectives:**

We investigated the feasibility and reproducibility of free-breathing motion-corrected multiple inversion time (multi-TI) pulsed renal arterial spin labelling (PASL), with general kinetic model parametric mapping, to simultaneously quantify renal perfusion (RBF), bolus arrival time (BAT) and tissue T_1_.

**Methods:**

In a study approved by the Health Research Authority, 12 healthy volunteers (mean age, 27.6 ± 18.5 years; 5 male) gave informed consent for renal imaging at 3 T using multi-TI ASL and conventional single-TI ASL. Glyceryl trinitrate (GTN) was used as a vasodilator challenge in six subjects. Flow-sensitive alternating inversion recovery (FAIR) preparation was used with background suppression and 3D-GRASE (gradient and spin echo) read-out, and images were motion-corrected. Parametric maps of RBF, BAT and T_1_ were derived for both kidneys. Agreement was assessed using Pearson correlation and Bland-Altman plots.

**Results:**

Inter-study correlation of whole-kidney RBF was good for both single-TI (*r*^2^ = 0.90), and multi-TI ASL (*r*^2^ = 0.92). Single-TI ASL gave a higher estimate of whole-kidney RBF compared to multi-TI ASL (mean bias, 29.3 ml/min/100 g; *p* <0.001). Using multi-TI ASL, the median T_1_ of renal cortex was shorter than that of medulla (799.6 ms vs 807.1 ms, *p* = 0.01), and mean whole-kidney BAT was 269.7 ± 56.5 ms. GTN had an effect on systolic blood pressure (*p* < 0.05) but the change in RBF was not significant.

**Conclusions:**

Free-breathing multi-TI renal ASL is feasible and reproducible at 3 T, providing simultaneous measurement of renal perfusion, haemodynamic parameters and tissue characteristics at baseline and during pharmacological challenge.

**Key points:**

*• Multiple inversion time arterial spin labelling (ASL) of the kidneys is feasible and reproducible at 3 T.*

*• This approach allows simultaneous mapping of renal perfusion, bolus arrival time and tissue T*_*1*_ *during free breathing.*

*• This technique enables repeated measures of renal haemodynamic characteristics during pharmacological challenge.*

**Electronic supplementary material:**

The online version of this article (10.1007/s00330-018-5628-3) contains supplementary material, which is available to authorized users.

## Introduction

The auto-regulatory feedback mechanisms that maintain renal blood flow (RBF) and glomerular filtration rate (GFR) over a wide range of haemodynamic pathophysiological conditions can be disturbed in metabolic and inflammatory disease states promoting the progression of nephropathy [1–3]. Measuring and interpreting altered renal perfusion at baseline and in response to physiological stressors would facilitate assessment of individuals at risk of kidney disease or its complications, and assist in the development of pharmacological interventions [4]. Measurement of renal perfusion currently relies on exogenous administration of contrast medium or clearance of radio-isotopes which have limited availability, may be contraindicated when renal function is impaired, and do not allow repeated real-time assessment during pharmacological challenge [5].

Magnetic resonance imaging (MRI) is emerging as a non-invasive approach for assessing renal structure and function including physiological parameters such as tissue perfusion, oxygenation, and water diffusion [6]. Arterial spin labelling (ASL) is a method that uses flowing blood as an endogenous contrast agent that has been widely used in neuro-imaging applications [7], and has also been shown to be feasible in healthy, transplanted and diseased kidneys [8]. ASL uses a radiofrequency (RF) pulse to magnetically label water protons in blood, so that they act as a diffusible tracer. Subtraction of the labelled images from control images allows perfusion maps to be quantified by fitting a kinetic model to the data [9]. In standard single-inversion time (TI) ASL techniques, a single inversion labelling pulse is applied, and a perfusion value is calculated using a simplified model which neglects variations in renal tissue T_1_ and makes assumptions concerning the bolus arrival characteristics and blood T_1_ [10, 11]. As the signal is dependent on the chosen TI, which specifies the interval between labelling and image acquisition, dynamically acquiring data at multiple-TIs would allow analysis using a general kinetic model that accounts for the effects of variable transit delays as well as tissue relation properties [12].

In this study, we investigated the feasibility and reproducibility of a free-breathing motion-corrected multi-TI pulsed renal ASL (PASL) sequence, with general kinetic model parametric mapping, enabling perfusion to be quantified with correction for bolus arrival time (BAT) and renal T_1_. We also assessed its potential to evaluate renal perfusion during pharmacological challenge. For this we used the vasodilator glyceryl trinitrate (GTN) which in animal models leads to a transient fall in systemic blood pressure but maintained kidney perfusion due to renal auto-regulatory mechanisms [13].

## Methods

### Subjects

This single-centre prospective observational and interventional study was approved by the Health Research Authority (17/EE/0068). Twelve healthy participants were recruited between March and July 2017. Standard published safety contraindications to MRI were applied [14]. No participants had known or suspected cardiovascular or renal disease, and none were pregnant. All participants gave written informed consent.

### Study protocol

A single-TI and multi-TI sequence were acquired consecutively in each participant. Reproducibility was assessed by repeating this protocol twice on the same day. In six participants, multi-TI ASL was used to assess the effect of administering a vasodilator. Heart-rate and blood pressure were monitored using an MRI-compatible system (TeslaM3; MIPM, Mammendorf, Germany). Following two consecutive baseline ASL measurements, 800 μg of GTN was administered sub-lingually. The first post-GTN image was acquired at 5 min, followed by three further measurements at 7-min intervals.

### Imaging protocol

**Fig. 1:**
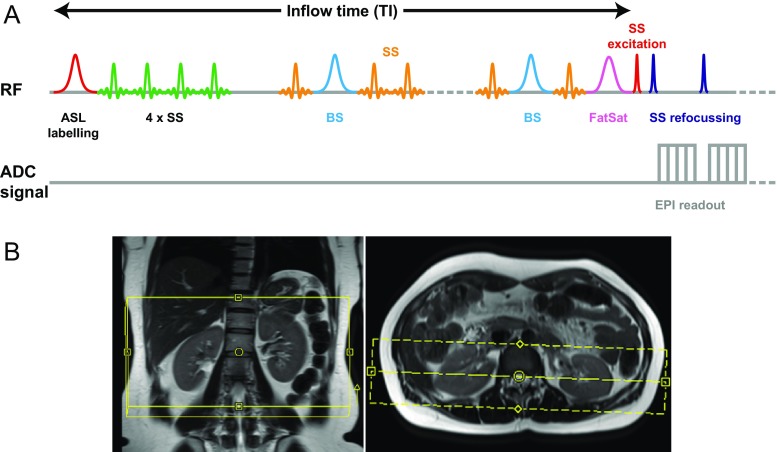
**A** Pulse sequence diagram for the multi-TI ASL sequence: radiofrequency (*RF*) preparation consists of an ASL labelling pulse (*red*) followed by four slice-selective (*SS*) pulses (*green*) on the labelling volume. A pair of background suppression (*BS*) pulses (*light blue*) occurs during a series of SS amplitude-modulated saturation pulses (*orange*) prior to a fat saturation prepulse (*pink*). The multi-inversion time readout begins with an SS excitation (*red*), followed by SS refocusing pulses (*dark blue*) with echo-planar imaging (*EPI*; *grey*). **B** Slice positioning of the imaging volume in renal ASL measurements. Aorta is not incorporated in the imaging volume to ensure proper labelling of blood flow into the region of interest

Supine imaging was performed on a 3T MAGNETOM Prisma (Siemens Healthcare, Erlangen, Germany) with XR gradients (80 mT/m at 200 T/m/s) using an 18-channel anterior coil and a six-channel posterior coil. Oblique coronal imaging of both kidneys was performed with the volume positioned to exclude the abdominal aorta. A proton density image (M_0_) was acquired as a reference for quantitative modelling, followed by control and label images. Each ASL acquisition employed a prototype flow sensitive alternating inversion recovery (FAIR) [15] preparation with a three-dimensional gradient and spin echo (3D-GRASE) [16] read-out (Fig. 1). The field of view (FOV) was 64 × 150 × 300 mm with a voxel size of 4 × 4.7 × 4.7 mm. All the k-space data for one TI were acquired at each excitation. The repetition (TR) and echo times (TE) for the single-TI protocol were TR/TE = 3,500/20.82 ms, with a receiver bandwidth (BW) of 2,298 Hz/pixel and 40 signal averages, giving a total acquisition time of 4 m 42 s; for the multi-TI protocol, the corresponding parameters were TR/TE = 3,500/16.88, BW = 3,720 Hz/pixel, 4 signal averages and 6 m 33 s. No partial-Fourier encoding, segmented acquisition or parallel imaging acceleration was used in any direction.

Background suppression (BS) was applied, consisting of an initial pre-saturation pulse, immediately after the labelling pulse, followed by a pair of separated adiabatic inversion pulses, each of 15.36 ms duration; this nulled signal from tissues with T_1_ of values 230 ms and 460 ms, working as a double-inversion preparation module. The timing of the inversion pulses was chosen such that the sum of the remaining tissue signals was significantly suppressed [17]. The BS pulses were followed by a delay of 30 ms, to avoid negative longitudinal magnetisation. The bolus length (BL) was limited to 800 ms using a train of modulated saturation pulses. A fat-saturation module was employed immediately before image acquisition, and a series of modulated saturation pulses was applied, anterior and posterior to the imaging volume, in order to limit the length of the labelled blood bolus and to reduce the effect of intra-vascular blood flowing into the volume of interest. Control and label images were corrected for motion by performing retrospective 2D elastic registration using proprietary vendor software. Images were acquired during free breathing. For the single-TI protocol, a T_1_ value for blood (T_1b_) of 1,250 ms was assumed for protons in water; the single TI was also 1,250 ms, whereas for the multi-TI protocol, there were 14 TIs, starting at 200 ms and increasing by uniform steps of 175 ms up to 2,475 ms. The range of TIs was chosen to sample both the onset of the renal perfusion time-course and the final signal decay.

### Parametric mapping

The perfusion weighted image (PWI) was constructed from control and label images, and the parameter maps (motion-corrected M_0_ and *f* for the single-TI protocol; also, BAT for the multi-TI acquisition) were calculated on-line from this, using the prior M_0_ image for signal calibration and image co-registration.

Voxel-wise single-TI perfusion values were calculated according to this formula (derived by means of simplifying assumptions from the full Buxton model) [18]:

$$ f=\frac{\lambda }{2TI\ }\ \frac{\varDelta M(TI)}{M_{0b}\ }\mathit{\exp}\frac{TI}{T_{1b}} $$

Perfusion values inferred from the multi-TI scans were calculated in accordance with the Buxton general kinetic model [19]:

$$ \varDelta M(t)= 2\ {M}_{0b}f\ \underset{0}{\overset{t}{\int }}c\left({t}^{\prime}\right)\ r\left(t-{t}^{\prime}\right)\ m\left(t-{t}^{\prime}\right)d{t}^{\prime } $$

For PASL, Buxton gives a delivery function *c*(*t*) which is non-zero within a fixed window, determined by the BAT Δt and the labelling pulse duration τ, modified by an inversion efficiency α and governed by T_1b_:

$$ {\displaystyle \begin{array}{l}c(t)=0,\kern0.5em 0\le t<\varDelta t\\ {}c(t)=\alpha {e}^{-\frac{t}{T_{1b}}},\kern0.5em \varDelta t\le t<\varDelta t+\tau \\ {}c(t)=0,\kern0.5em \varDelta t+\tau \le t\end{array}} $$

The residue (or venous clearance) function *r*(*t*) is governed, in turn, by the exchange of protons between water and tissue:

$$ r(t)={e}^{-\frac{ft}{\lambda }} $$

Finally, the relaxation function is controlled by the T_1_ of tissue:

$$ m(t)={e}^{-\frac{t}{T_1}} $$

The new terms introduced here are:

**Table:** 

*∆M*	the difference between selective and non-selective inversion images
*M*_*0b*_	equilibrium magnetization (containing blood spins only)
*f*	renal blood flow, expressed as ml/min/100 g of tissue
*T*_*1b*_	longitudinal relaxation time of blood
*T*_*1*_	longitudinal relaxation time of tissue
*α*	inversion efficiency (given as 0.98)
*Δt*	bolus arrival time
*λ*	the tissue-blood partition coefficient of water, given as 0.9 ml/g
*t*	in-flow or inversion time (between the end of the labelling pulse and the beginning of read-out)
*t′*	a dummy variable, such that 0 ≤ *t′* ≤ *t*

### Kidney segmentation

Whole kidneys were manually segmented slice by slice in MATLAB 2016b (MathWorks, Natick, MA, USA). For the avoidance of partial-volume effects, only the central eight sections of each 16-section 3D image were used. In order to allow a consistent comparison between single-TI and multi-TI studies, the cortex was considered as the outer 3 voxels bounded by the renal segmentation. We also demonstrated segmentation of the cortex from the medulla on multi-TI datasets by propagating a mask, obtained by applying a threshold to the T_1_ map histogram, to the perfusion map [8]. We adopted a semi-automated approach where an observer interactively set a T_1_ threshold while viewing the resulting segmentation on the 3D volume of images.

### Statistical analysis

Model fitting and parametric mapping were performed in MATLAB 2016b (MathWorks) and statistical analysis in IBM SPSS version 22 (IBM, Armonk, NY, USA). Categorical variables were expressed as percentages and continuous variables as mean ± standard deviation (SD). The correlation between repeated measurements was assessed using Pearson’s coefficient with major axis regression. The agreement between methods was assessed using Bland-Altman plots with 95% confidence intervals (CI) for the mean difference and 95% CI for the limits of agreement. Continuous variables were compared with the Wilcoxon signed-rank test. The difference in means of repeated observations in the interventional study was assessed with a repeated-measures analysis of variance (ANOVA). In all tests, a *p* value <0.05 was taken as significant.

## Results

### Study participant demographics

All 12 subjects successfully completed the protocol. All data sets were analysed and included in the final analysis. There were five men (age, 26.8 ± 7.3 years; weight, 73.8 ± 10.6 kg; height, 172 ± 1.8 cm) and seven women (age, 28.5 ± 5.6 years; weight, 66.2 ± 6.5 kg; height, 170.6 ± 10.3 cm).

### ASL parameters

**Fig. 2:**
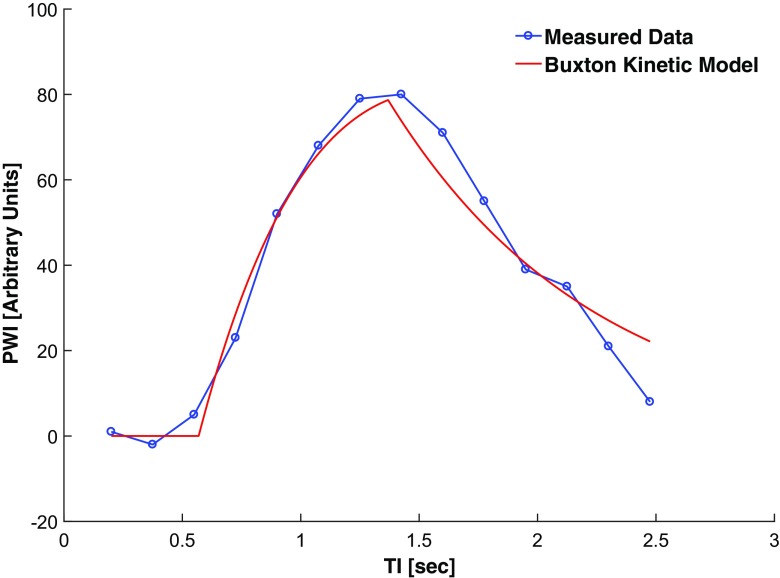
An example of an observed single-voxel multi-TI time-course of renal perfusion at 14 TIs, with a fitted Buxton kinetic model (*red*)

**Fig. 3:**
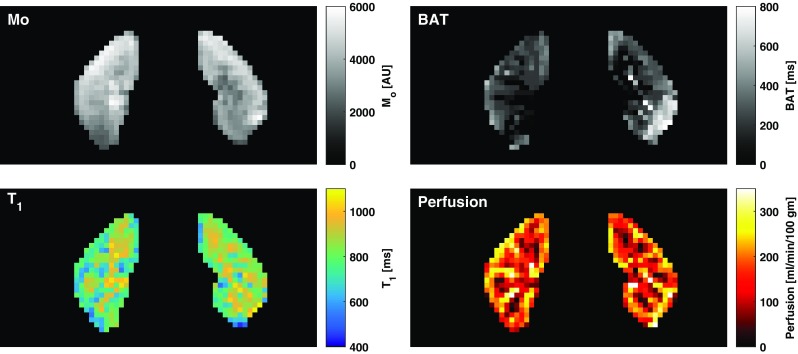
Parametric mapping using fitting of multi-TI ASL data to a general kinetic model. Coronal images of both kidneys are shown with M_0_, T_1_, BAT and perfusion maps

**Fig. 4:**
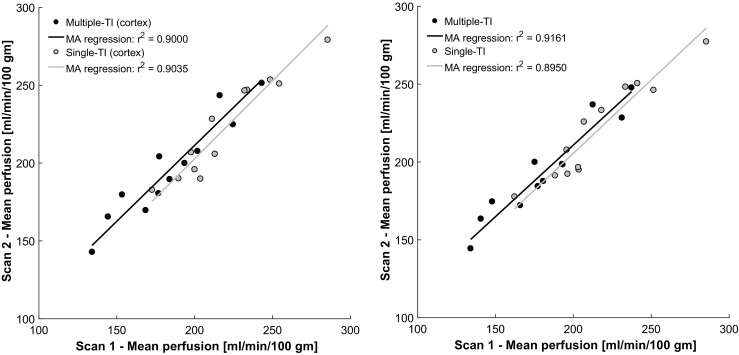
Scatterplot with major axis (MA) regression of inter-study perfusion measurement of cortex and whole kidney using single-TI and multi-TI ASL

**Fig. 5:**
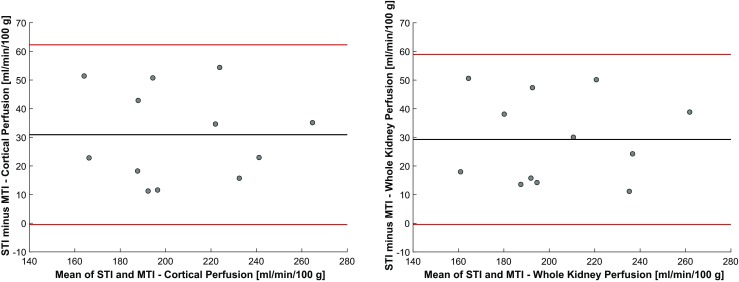
Bland-Altman comparison of single-TI and multi-TI ASL for cortical and whole kidney perfusion measurement showing mean bias and limits of agreement

**Fig. 6:**
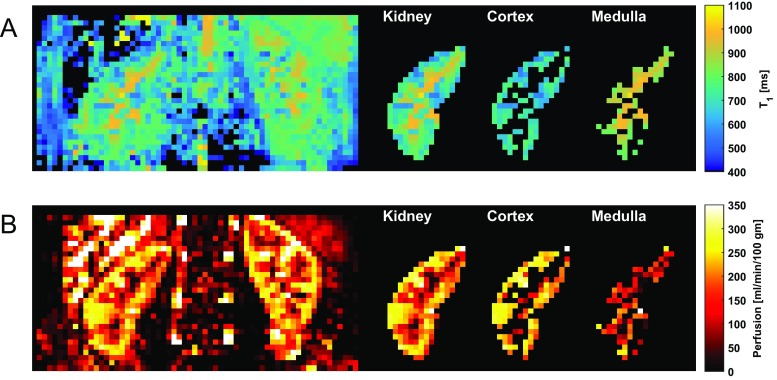
Parametric mapping of multi-TI ASL data enables cortex to be differentiated from medulla by the difference in T_1_ (**a**), and for this to be propagated to the perfusion map for anatomic segmentation (**b**). The corresponding histogram and threshold is shown in Supplementary Fig. 2

Examples of the model fitting and parametric mapping of multi-TI ASL (using the outer 3 voxels to define renal cortex) are shown in Figs. 2 and 3, and Supplementary Video 1, with derived variables summarised in Table 1. Inter-study correlation and the agreement between single and multi-TI methods are shown in Figs. 4 and 5. An example of semi-automated segmentation (using T_1_ thresholding to define renal cortex) is shown in Fig. 6, with the corresponding histogram in Supplementary Fig. 1, and the resulting derived data given in Supplementary Table 1.

**Table 1:** Parameters derived from single and multi-TI renal ASL in 12 healthy volunteers using the outer 3 voxels to define renal cortex

	Scan 1		Scan 2	
	Multi-TI	Single-TI	Multi-TI	Single-TI
*Perfusion (ml/min/100 g):*				
Whole kidney	182.39 ± 33.24	215.47 ± 32.84	194.70 ± 30.68	220.16 ± 31.07
Cortex	184.84 ± 32.88	220.20 ± 31.72	196.61 ± 32.12	223.05 ± 31.78
Medulla	168.49 ± 37.87	179.16 ± 37.39	182.66 ± 34.96	188.40 ± 39.76
*Bolus arrival time (ms):*				
Whole kidney	262.45 ± 51.44		277.01 ± 62.48	
Cortex	290.77 ± 70.07		302.74 ± 71.75	
Medulla	184.21 ± 37.93		196.82 ± 54.84	
*T*_*1*_ *(ms):*				
Whole kidney	774.02 ± 30.69		781.59 ± 28.64	
Cortex	786.17 ± 31.33		794.84 ± 32.28	
Medulla	799.32 ± 38.25		811.34 ± 25.49	

Values are mean ± standard deviation, averaged over both kidneys

Using multi-TI ASL, averaged over both kidneys, mean RBF was 188.54 ± 31.91 ml/min/100 g in the whole kidney and 190.72 ± 32.35 ml/min/100 g in the cortex. Inter-study correlation (*r*^2^) was 0.91 for whole kidney perfusion and 0.90 for cortical perfusion. The T_1_ of the renal cortex was shorter than that of the medulla (799.61 vs 807.11 ms, *p* = 0.01). Mean BAT in the whole kidney was 269.73 ± 58.43 ms.

Using single-TI ASL, averaged over both kidneys, mean RBF was 217.81 ± 31.36 ml/min/100 g in the whole kidney and 221.62 ± 31.08 ml/min/100 g in the cortex. The mean bias between single-TI and multi-TI of whole kidney RBF was 29.27 ml/min/100 g [CI, (19.65, 38.89) ml/min/100 g; limits of agreement, (-0.41, 58.95) ml/min/100 g, *p* < 0.001]. The mean bias between single-TI and multi-TI of cortical RBF was 30.90 ml/min/100 g [CI, (20.73, 41.08) ml/min/100 g; limits of agreement, (-0.48, 62.28) ml/min/100 g, *p* < 0.001]. Using the single-TI technique inter-study correlation (*r*^2^) was 0.89 for whole kidney perfusion and 0.90 for cortical perfusion.

### GTN challenge

GTN administration was well tolerated by all participants. Mean systolic (*p* = 0.02) and diastolic blood pressure (*p* = 0.01) differed significantly between time points, but the effect on RBF (*p* = 0.33) and BAT (*p* = 0.13) was not significant (Supplementary Fig. 2).

## Discussion

Free-breathing multi-TI ASL acquired at 3 T allows simultaneous measurement of RBF, BAT and tissue T_1_ using a general kinetic model for parameter fitting. Such an approach obviates the need for the parameter assumptions of single-TI ASL, is well-tolerated in healthy subjects and is highly reproducible. This technique enables repeated measures of renal haemodynamic characteristics during pharmacological challenge and offers a robust method for assessing renal physiological mechanisms over time.

Respiratory motion has posed a significant challenge in renal ASL due to the potential for mis-registration of pixels between temporally-adjacent labelled and control images—which is compounded by the longer readouts required for multi-TI ASL. Previous work has relied on acquiring data at multiple inversion times during separate breath-holds at 3 T [20, 21], or using prospective respiratory gating at 1.5 T [22, 23]. The disadvantage of prospective gating is that it prolongs acquisition time unnecessarily if separation of respiratory phases is not required. Alternatively, sequential breath-hold acquisitions may be up to 24 s [20], leading to a technical failure rate of approximately 8% [24, 25]. Whilst affine registration can adjust for bulk motion [26], our non-linear elastic model may be more suited to the non-rigid motion typical of abdominal imaging and enabled a time-efficient free-breathing acquisition for multi-TI ASL.

Our ASL values for cortical renal perfusion are in agreement with those obtained at 3 T using breath-hold multi-TI sequences: 151 ± 37 to 215 ± 65 ml/min/100 g [20, 21], but even in healthy volunteers at this field strength single-TI values range between 229 ± 41 to 327 ± 63 ml/min/100 g [10, 11, 26]. This variation can be attributed to different acquisition schemes, kinetic models and study populations. In this regard, multi-TI ASL could be advantageous since it takes delivery, clearance and relaxation effects fully into account to better represent the dynamics of renal blood flow. The longer T_1_ of tissues at high field strength also allows for longer post-labelling delays and therefore higher signal in PWI [27]. Previous studies using multiple post-labelling delays have demonstrated good correlation between ASL-derived parameters and dynamic contrast-enhancement (DCE) [20], although more modest agreement was observed with ^99m^Tc-MAG3 scintigraphy—suspected to be a consequence of respiratory motion [25]. Variations in renal ATT have been observed between young and old age groups, and multi-TI ASL enables patient-specific modelling of RBF [25]. Beyond correction of absolute RBF estimates, the value of BAT as a parameter of renal haemodynamic state is unknown and worthy of further investigation.

Kidney interstitial fibrosis is a histological finding that represents a common final pathway in many renal diseases and T_1_ values correlate well with fibrosis and inflammation in both animal models and humans [28]. Multi-TI ASL enables renal T_1_ values to be modelled as part of a multi-parametric assessment of renal function and avoids the need to acquire and co-register a separate renal T_1_ mapping sequence [11]. T_1_ maps that are spatially aligned with other parametric maps enable an objective and consistent segmentation of cortex from medulla, which is important as altered cortico-medullary physiology is a characteristic of renal insufficiency [29]. Our T_1_ values for renal cortex and medulla were lower than previously-reported data at 3 T, using an inversion recovery method, with less differentiation between these tissues.[30] A plausible explanation would be variation in effective T_1_* due to the influence of different read-out pulses on recovery of longitudinal magnetisation. Recently, a T_1_ mapping phantom has been proposed for cardiac MRI to ensure measurement stability over time at individual sites across vendors, software versions and imaging sequences [31], which could be adapted to the clinically relevant ranges overserved in the kidney. Otherwise, if quantitative values are reported reference ranges obtained from healthy volunteers at each imaging centre may be necessary [32].

Previous studies have shown the sensitivity of ASL to map dynamic changes in renal perfusion during administration of adenosine [33], protein load [34] and furosemide [35]. GTN is known to be a potent venodilator with the acute effect mainly being a reduction of systolic blood pressure due to an increase in arterial compliance and a decrease in venous return resulting in reflex tachycardia [36]. In contrast to other smooth muscle vasodilators, renal autoregulation maintains stable arterial perfusion during GTN administration in animal models [13]. We observed a typical haemodynamic response to sublingual GTN but the effect on RBF was not significant consistent with stable blood flow despite a fluctuation in perfusion pressure. This experiment shows as a proof-of-principle how simultaneous dynamic assessment of multiple renal perfusion and tissue characteristics (i.e. BAT, RBF, T_1_) can be readily performed during multi-TI renal ASL.

Our study has limitations. We used a relatively small sample of young healthy adults and this may not be representative of broader populations and those with renal impairment. However, the acquisition technique is not dependent on breath-hold capability and would be expected to be well-tolerated in patient groups. We did not have a “gold standard” of renal perfusion to compare with as administration of either gadolinium-based contrast agents or radio-tracers to healthy subjects is avoided if possible. Future work on quantitative evaluation of ASL could be validated against phantoms that simulate capillary blood flow (Qasper; Gold Standard Phantoms, London, UK).

In conclusion, free-breathing multi-TI renal ASL is a reproducible and well-tolerated approach for simultaneous parametric mapping of kidney perfusion and tissue characteristics at 3 T.

## Electronic supplementary material

ESM 1 (PDF 495 kb)

Supplementary Video 1 Single coronal section of the kidneys showing renal perfusion values derived from multi-TI ASL displayed at real-time speed: first TI = 200 ms, with 13 subsequent TIs spaced at 175-ms intervals (AVI 892 kb).

## Abbreviations

ASL: Arterial spin labelling
ATT: Arterial transit time
BAT: Bolus arrival time
BL: Bolus length
BW: Bandwidth
BS: Background suppression
FAIR: Flow-sensitive alternating inversion recovery
GFR: Glomerular filtration rate
GRASE: Gradient and spin echo
GTN: Glyceryl trinitrate
M_0_: Proton density image
PASL: Pulsed arterial spin labelling
RBF: Renal blood flow
T_1b_: T_1_ value of blood

## References

[1] Carlstrom M, Wilcox CS, Arendshorst WJ (2015). Renal autoregulation in health and disease. Physiol Rev.

[2] Liu R (2017). The real culprit behind diabetic nephropathy: impaired renal autoregulation?. Physiol Rep.

[3] Ge Y, Fan F, Didion SP, Roman RJ (2017). Impaired myogenic response of the afferent arteriole contributes to the increased susceptibility to renal disease in Milan normotensive rats. Physiol Rep.

[4] Mongardon N, Dyson A, Singer M (2009). Pharmacological optimization of tissue perfusion. Br J Anaesth.

[5] Tan H, Koktzoglou I, Prasad PV (2014). Renal perfusion imaging with two-dimensional navigator gated arterial spin labeling. Magn Reson Med.

[6] Grenier N, Merville P, Combe C (2016). Radiologic imaging of the renal parenchyma structure and function. Nat Rev Nephrol.

[7] Lanzman B, Heit JJ (2017). Advanced MRI measures of cerebral perfusion and their clinical applications. Top Magn Reson Imaging.

[8] Cox EF, Buchanan CE, Bradley CR (2017). Multiparametric renal magnetic resonance imaging: validation, interventions, and alterations in chronic kidney disease. Front Physiol.

[9] Winter JD, St Lawrence KS, Cheng HL (2011). Quantification of renal perfusion: comparison of arterial spin labeling and dynamic contrast-enhanced MRI. J Magn Reson Imaging.

[10] Gillis KA, McComb C, Foster JE (2014). Inter-study reproducibility of arterial spin labelling magnetic resonance imaging for measurement of renal perfusion in healthy volunteers at 3 Tesla. BMC Nephrol.

[11] Gillis KA, McComb C, Patel RK (2016). Non-Contrast Renal Magnetic Resonance Imaging to Assess Perfusion and Corticomedullary Differentiation in Health and Chronic Kidney Disease. Nephron.

[12] Johnston ME, Lu K, Maldjian JA, Jung Y (2015). Multi-TI arterial spin labeling MRI with Variable TR and Bolus Duration for Cerebral Blood Flow and Arterial Transit Time Mapping. IEEE Trans Med Imaging.

[13] Ogawa N, Ono H (1986). Different effects of various vasodilators on autoregulation of renal blood flow in anesthetized dogs. Jpn J Pharmacol.

[14] Shellock FG (2017) Reference manual for magnetic resonance safety, implants, and devices: 2017 edition. Biomedical Research Publishing Group, Playa Del Rey

[15] Kim SG (1995). Quantification of relative cerebral blood flow change by flow-sensitive alternating inversion recovery (FAIR) technique: application to functional mapping. Magn Reson Med.

[16] Gunther M, Oshio K, Feinberg DA (2005). Single-shot 3D imaging techniques improve arterial spin labeling perfusion measurements. Magn Reson Med.

[17] Garcia DM, Duhamel G, Alsop DC (2005). Efficiency of inversion pulses for background suppressed arterial spin labeling. Magn Reson Med.

[18] Wang J, Licht DJ, Jahng GH (2003). Pediatric perfusion imaging using pulsed arterial spin labeling. J Magn Reson Imaging.

[19] Buxton RB, Frank LR, Wong EC, Siewert B, Warach S, Edelman RR (1998). A general kinetic model for quantitative perfusion imaging with arterial spin labeling. Magn Reson Med.

[20] Conlin CC, Oesingmann N, Bolster B, Huang Y, Lee VS, Zhang JL (2017). Renal plasma flow (RPF) measured with multiple-inversion-time arterial spin labeling (ASL) and tracer kinetic analysis: Validation against a dynamic contrast-enhancement method. Magn Reson Imaging.

[21] Kim DW, Shim WH, Yoon SK (2017). Measurement of arterial transit time and renal blood flow using pseudocontinuous ASL MRI with multiple post-labeling delays: Feasibility, reproducibility. and variation. J Magn Reson Imaging.

[22] Cutajar M, Thomas DL, Banks T, Clark CA, Golay X, Gordon I (2012). Repeatability of renal arterial spin labelling MRI in healthy subjects. MAGMA.

[23] Cutajar M, Thomas DL, Hales PW, Banks T, Clark CA, Gordon I (2014). Comparison of ASL and DCE MRI for the non-invasive measurement of renal blood flow: quantification and reproducibility. Eur Radiol.

[24] Petersen ET, Zimine I, Ho YC, Golay X (2006). Non-invasive measurement of perfusion: a critical review of arterial spin labelling techniques. Br J Radiol.

[25] Shimizu K, Kosaka N, Fujiwara Y (2017). Arterial transit time-corrected renal blood flow measurement with pulsed continuous arterial spin labeling MR Imaging. Magn Reson Med Sci.

[26] Wu WC, Su MY, Chang CC, Tseng WY, Liu KL (2011). Renal perfusion 3-T MR imaging: a comparative study of arterial spin labeling and dynamic contrast-enhanced techniques. Radiology.

[27] Wang J, Zhang Y, Wolf RL, Roc AC, Alsop DC, Detre JA (2005). Amplitude-modulated continuous arterial spin-labeling 3.0-T perfusion MR imaging with a single coil: feasibility study. Radiology.

[28] Friedli I, Crowe LA, Berchtold L (2016). New magnetic resonance imaging index for renal fibrosis assessment: a comparison between diffusion-weighted imaging and T1 mapping with histological validation. Sci Rep.

[29] Lee VS, Kaur M, Bokacheva L (2007). What causes diminished corticomedullary differentiation in renal insufficiency?. J Magn Reson Imaging.

[30] de Bazelaire CM, Duhamel GD, Rofsky NM, Alsop DC (2004). MR imaging relaxation times of abdominal and pelvic tissues measured in vivo at 3.0 T: preliminary results. Radiology.

[31] Captur G, Gatehouse P, Keenan KE et al (2016) A medical device-grade T1 and ECV phantom for global T1 mapping quality assurance—the T1 Mapping and ECV Standardization in cardiovascular magnetic resonance (T1MES) program. J Cardiovasc Magn Reson 18(58) 10.1186/s12968-016-0280-z PMC5034411 27660042

[32] Messroghli DR, Moon JC, Ferreira VM (2017). Clinical recommendations for cardiovascular magnetic resonance mapping of T1, T2, T2* and extracellular volume: A consensus statement by the Society for Cardiovascular Magnetic Resonance (SCMR) endorsed by the European Association for Cardiovascular Imaging (EACVI). J Cardiovasc Magn Reson.

[33] Tan H, Thacker J, Franklin T, Prasad PV (2015). Sensitivity of arterial spin labeling perfusion MRI to pharmacologically induced perfusion changes in rat kidneys. J Magn Reson Imaging.

[34] He X, Aghayev A, Gumus S, Ty Bae K (2014). Estimation of single-kidney glomerular filtration rate without exogenous contrast agent. Magn Reson Med.

[35] Wang J, Zhang Y, Yang X (2012). Hemodynamic effects of furosemide on renal perfusion as evaluated by ASL-MRI. Acad Radiol.

[36] Kawakami H, Sumimoto T, Hamada M (1995). Acute effect of glyceryl trinitrate on systolic blood pressure and other hemodynamic variables. Angiology.

