# Supplementary material for: Motion-corrected multiparametric renal arterial spin labelling at 3 T: reproducibility and effect of vasodilator challenge
Source: Eur Radiol. 2018 Jul 10;29(1):232–40. doi: 10.1007/s00330-018-5628-3 (PMC6291439; doi:10.1007/s00330-018-5628-3)
Supplement: Supplementary file 1 — (PDF 495 kb) [file 330_2018_5628_MOESM1_ESM.pdf]

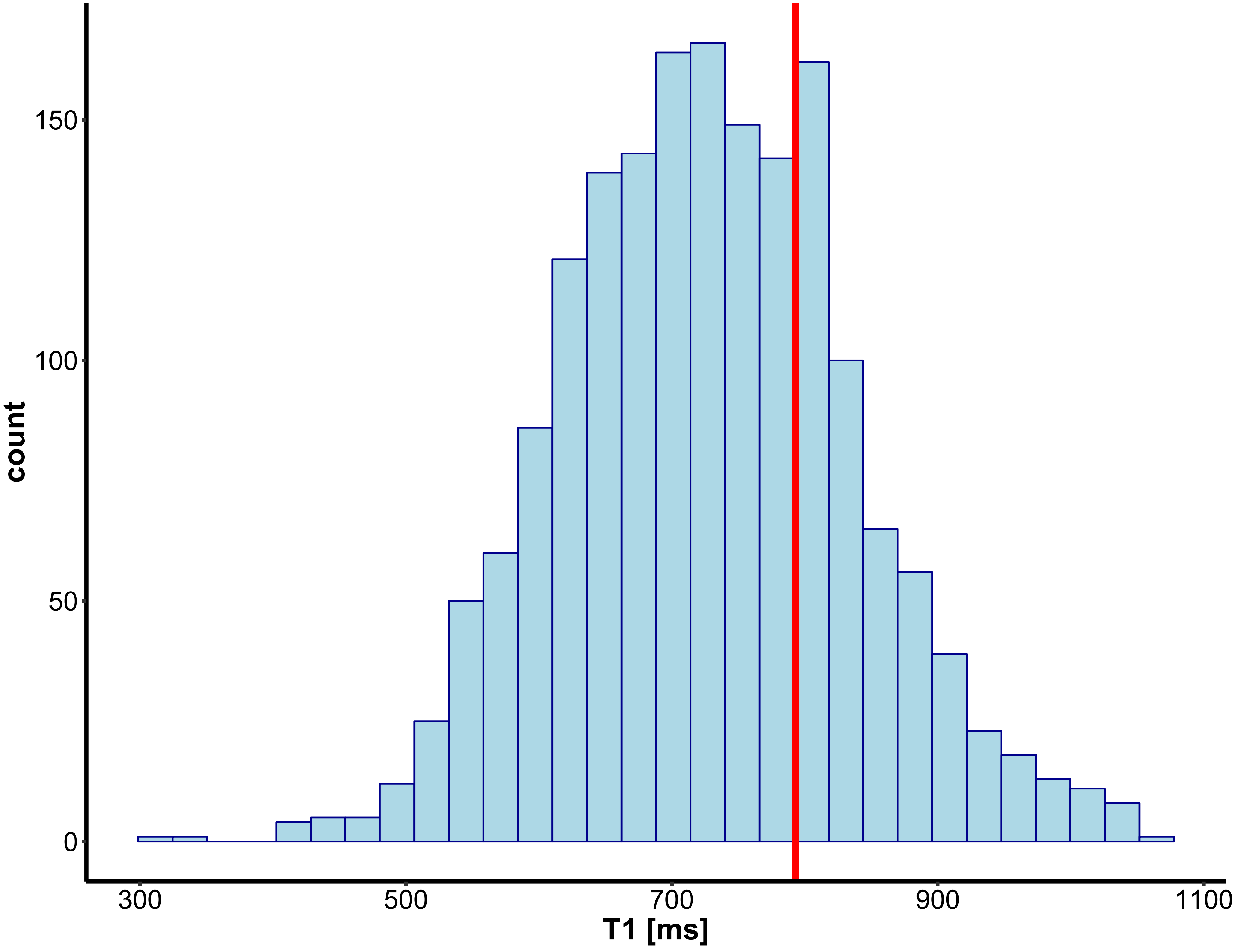

Supplementary Figure 1. Histogram of renal T1 values, for the subject shown in Figure 6, showing the threshold chosen to segment cortex from medulla.

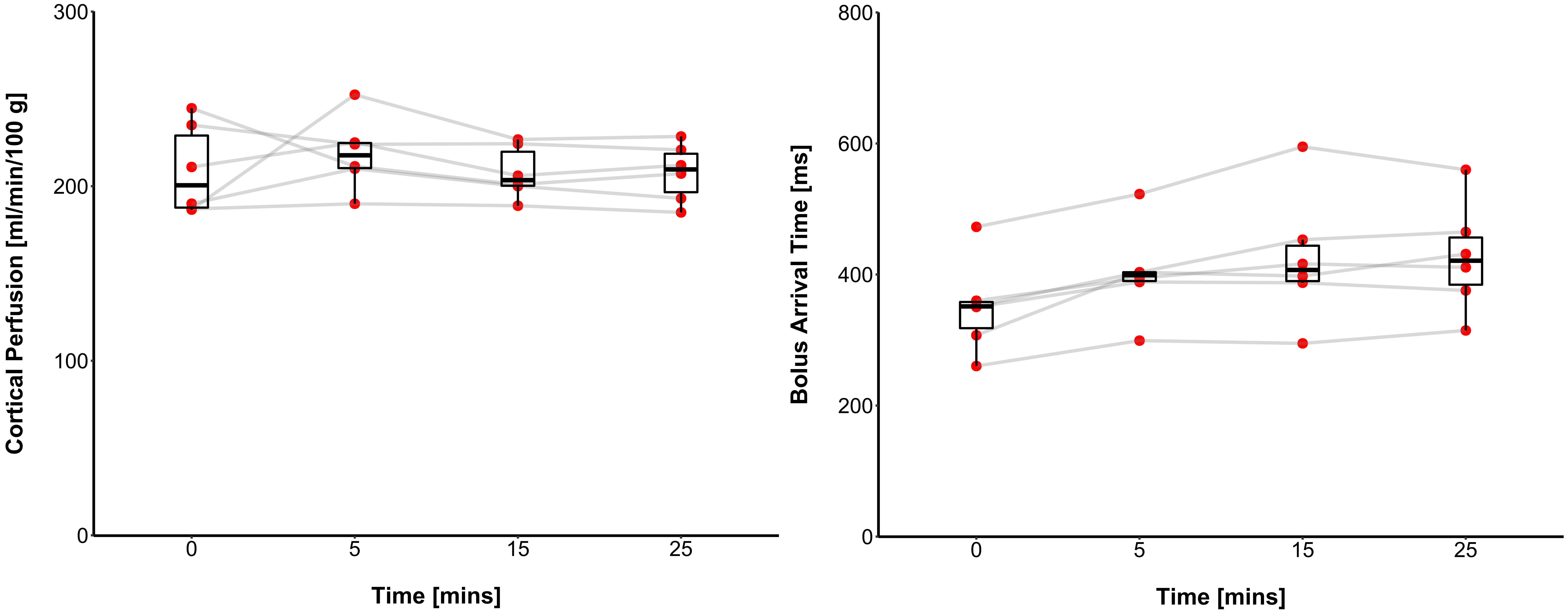

Supplementary Figure 2. Tukey box-plots with data points for each subject (n=6) showing cortical perfusion and bolus arrival time at baseline and following the administration of GTN acquired using multi-TI ASL.

Supplementary Table 1. A summary of parameters acquired in this study, listed as mean ± standard deviation, averaged over both kidneys with segmentation by means of T_1 thresholding.

	**Scan 1**		**Scan 2**	
	Multi-TI	Single-TI	Multi-TI	Single-TI
**Perfusion [ml/min/100 gm]:**				
Whole kidney	182.39 ± 33.24	215.47 ± 32.84	194.70 ± 30.68	220.16 ± 31.07
Cortex	207.90 ± 36.01		222.09 ± 32.47	
Medulla	142.65 ± 28.09		154.21 ± 27.49	
**Bolus Arrival Time [ms]:**				
Whole kidney	262.45 ± 51.44		277.01 ± 62.48	
Cortex	254.36 ± 36.79		270.57 ± 49.57	
Medulla	266.69 ± 70.22		281.55 ± 72.87	
**T_1 [ms]:**				
Whole kidney	774.02 ± 30.69		781.59 ± 28.64	
Cortex	708.80 ± 34.47		718.41 ± 29.18	
Medulla	870.78 ± 24.46		872.05 ± 24.49	
